# Lamellipodia and Membrane Blebs Drive Efficient Electrotactic Migration of Rat Walker Carcinosarcoma Cells WC 256

**DOI:** 10.1371/journal.pone.0149133

**Published:** 2016-02-10

**Authors:** Jolanta Sroka, Izabela Krecioch, Eliza Zimolag, Slawomir Lasota, Monika Rak, Sylwia Kedracka-Krok, Pawel Borowicz, Marta Gajek, Zbigniew Madeja

**Affiliations:** 1 Department of Cell Biology, Faculty of Biochemistry, Biophysics and Biotechnology, Jagiellonian University, Gronostajowa 7, 30–387, Krakow, Poland; 2 Department of Physical Biochemistry, Faculty of Biochemistry, Biophysics and Biotechnology, Jagiellonian University, Gronostajowa 7, 30–387, Krakow, Poland; 3 Malopolska Centre of Biotechnology, Jagiellonian University, Gronostajowa 7b, 30–387, Krakow, Poland; BloodCenter of Wisconsin, UNITED STATES

## Abstract

The endogenous electric field (EF) may provide an important signal for directional cell migration during wound healing, embryonic development and cancer metastasis but the mechanism of cell electrotaxis is poorly understood. Additionally, there is no research addressing the question on the difference in electrotactic motility of cells representing various strategies of cell movement—specifically blebbing vs. lamellipodial migration. In the current study we constructed a unique experimental model which allowed for the investigation of electrotactic movement of cells of the same origin but representing different modes of cell migration: weakly adherent, spontaneously blebbing (BC) and lamellipodia forming (LC) WC256 cells. We report that both BC and LC sublines show robust cathodal migration in a physiological EF (1–3 V/cm). The directionality of cell movement was completely reversible upon reversing the field polarity. However, the full reversal of cell direction after the change of EF polarity was much faster in the case of BC (10 minutes) than LC cells (30 minutes). We also investigated the distinct requirements for Rac, Cdc42 and Rho pathways and intracellular Ca^2+^ in electrotaxis of WC256 sublines forming different types of cell protrusions. It was found that Rac1 is required for directional movement of LC to a much greater extent than for BC, but Cdc42 and RhoA are more crucial for BC than for LC cells. The inhibition of ROCK did not affect electrotaxis of LC in contrast to BC cells. The results also showed that intracellular Ca^2+^ is essential only for the electrotactic reaction of BC cells. Moreover, inhibition of MLCK and myosin II did not affect the electrotaxis of LC in contrast to BC cells. In conclusion, our results revealed that both lamellipodia and membrane blebs can efficiently drive electrotactic migration of WC 256 carcinosarcoma cells, however directional migration is mediated by different signalling pathways.

## Introduction

Cell migration is a highly integrated multistep process that plays a critical role in a variety of normal physiological events and in many diseases. There are general similarities in strategies of cell migration across cell types. However, different modes of cell migration dependent on cell type and the surrounding environment were described. Cells can move either individually (ameboid or mesenchymal movement) or collectively as multicellular cohorts [1]. Ameboid and mesenchymal movement seem to be extremes of a continuum of various strategies of cell movement [2]. At one end, ameboid movement is characterised by gliding and rapid migration of weakly adherent rounded or ellipsoid cells that lack stress fibers. At the other extreme, mesenchymal cell migration is described as the movement of polarized fibroblast-like cells, reliant on protease-dependent degradation of the ECM, formation of lamellipodia and strong, integrin-dependent contacts [1, 2]. One of the described subtypes of ameboid movement is referred to as blebbing motility [1]. Blebs are cellular protrusions which expand by hydrostatic pressure generated in the cytoplasm by the contractile actomyosin cortex and are initially free of actin filaments. Several cell types, from amoebae to mammalian embryonic and tumour cells, can use this strategy of cell migration. It should be noted that there is a fundamental difference between bleb formation and the mechanisms of lamellipodia expansion [3, 4]. In contrast to bleb formation, a central role in lamellipodium expansion is played by actin polymerization which drives the protrusion of the cell membrane. However, if blebbing migration is considered an alternative to lamellipodia-driven cell motility, an important question arises concerning the mechanisms by which bleb formation is biased toward the leading edge of the cell. Although the mechanism of regulation of blebbing cells’ directional movement is unclear, it was observed that bleb location appears to be controlled directly by chemotactic gradients [5, 6, 7, 8, 9]. This suggests that the same chemotactic factors may induce formation of different types of cell protrusions to prompt directional movement. Moreover, dynamic transitions between amoeboid and mesenchymal migration [10] and switching between bleb or lamellipodia formation during cell migration were observed [4]. However, how chemoattractants induce different types of cell protrusions is unclear, and the mechanism leading from extracellular signal to polarization of blebbing activity is also not fully understood. Additionally, directional cell migration is induced not only by chemoattractants but also by physical cues like substrate anisotropy (contact guidance) and electric fields (electrotaxis) [11, 12, 13, 14].

The presence of endogenous electric fields (EFs) within extracellular spaces has been known for more than 150 years, however the significance of EFs for numerous physiological processes has only recently been confirmed by several modern techniques. Such electric fields are generated by directional transport of ions across various epithelia as a result of the polarized distribution of ion channels and pumps [15]. For instance, direct current electric fields (dcEFs) between 0.4–2 V/cm were measured near mammalian skin wounds [16, 17]. Accumulating evidence suggests an important role for electric signals in directing cell migration in wound healing, embryonic development and cancer metastasis [12, 15, 18]. A variety of motile cells respond to dcEFs by changing the orientation of their movement and by active directional migration towards the cathode or anode, a phenomenon called electrotaxis or galvanotaxis [18, 19, 20, 21].

The mechanisms of electrotaxis are extensively investigated but poorly understood. One plausible hypothesis assumes that the electrostatic or electroosmotic forces redistributes charged components of the cell membrane, including receptors for chemoattractants. The increase in density of membrane receptors on the cathode (or anode) facing side of the cell is responsible for asymmetric signaling and directional motility. In this model the molecular mechanism of induction of directional movement is the same in chemotaxis and electrotaxis (i.e. asymmetrical activation of receptors of chemoattractants), however in chemotaxis it is caused by a gradient of chemoattractants whereas in electrotaxis by the redistribution of receptors in EFs [18, 22]. This mechanism may be responsible for long-lasting electrotactic effects, however it does not explain the dynamics of the first, very fast reactions of cells to EFs [12, 13]. Electrotactic movement was described for cells representing various modes of cell movement, for example fibroblasts, keratinocytes, neutrophils and neural crest cells [23, 24, 25, 26]. Interestingly, it was reported that a nanosecond pulsed electric field (10-kV/cm pulses delivered at 10–20 Hz) induced formation of blebs in U937 cells but no systematic research has been conducted on the effects of physiological dcEFs on migration of blebbing cells and on a comparison of bleb- and lamellipodia-driven electrotaxis [27, 28]. An increasing number of studies have shown that moving cells exhibit heterogeneity in migration strategy. It was demonstrated that cells with high activity of Rac1, a key regulator of actin polymerization, often display mesenchymal motility, while high Rho activity, promoting actomyosin contractility, correlates with ameboid migration [3]. Therefore, it is possible that cells using hydrostatic pressure for generating cell protrusions (blebbing cells) represent a different mechanism of reaction to electric fields than lamellipodia forming cells, especially in relation to Rho GTPases which are the central signalling hubs through which diverse input signals important for cell migration are funneled [29]. Moreover, the important role of Rho signaling in electrotaxis was demonstrated directly by Rajnicek et al. [30]. On the other hand, contractility which is crucial for bleb formation and is primarily regulated by activity of non-muscle myosin II, is not only controlled by the Rho pathway but also by changes in intracellular Ca^2+^ concentration [31]. Interestingly, in most investigated cells Ca^2+^ dependence of electrotaxis has been observed [32]. In our previous paper [33] we reported that adherent WC 256 cells show efficient electrotactic migration. In the current study we constructed a unique experimental model allowing for the investigation of electrotactic movement of cells representing different modes of cell migration. We performed a quantitative analysis of electrotactic movement of two adherent sublines of Walker carcinosarcoma cell line WC 256, representing blebbing or lamellipodial migration. Finally, after initial investigation of the effect of electric fields on directional movement of WC 256 cells, we revealed the distinct requirements for Rho and Rac GTPases and Ca^2+^ ions in guidance by electric fields of WC 256 sublines forming different types of cell protrusions.

## Materials and Methods

### Materials

The chemicals were purchased as follows: RPMI-1640 medium, blebbistatin, ML-7, formic acid (FA) (Sigma); Rac1 inhibitor (NSC23766), Cdc42 inhibitor III (ZCL278), Rho inhibitor (Rhosin), ROCK inhibitor (Y-27632), BAPTA-AM (Calbiochem); fetal calf serum, Opti-MEM® I (Gibco); Lipofectamine 2000 Reagent (Invitrogen); Colorimetric G-LISA activity assay kits (Cytoskeleton, Inc., catalog numbers BK124, BK127, and BK128); urea, iodoacetamide (BioShop), ammonium bicarbonate (Fluka). TFA (trifluoroacetic acid), acetonitrile (ACN) (JT Baker, USA).

### Cell Culture

The adherent, lamellipodia forming subline of Walker carcinosarcoma WC256 cells (LC) was obtained by continuous culture of cells initially growing in a suspension as described previously [34]. Additionally, in the present study we obtained weakly adherent, spontaneously blebbing WC256 cells (BC) by culturing cells growing in suspension in the same flask without shaking for 1–2 weeks. Cells were cultured in RPMI-1640 medium supplemented with 5% fetal calf serum (FCS), 100 IU/ml penicillin and 10 μg/ml streptomycin in humidified atmosphere with 5% CO_2_ at 37°C.

### Cell Shape Analysis

The parameters characterizing cell morphology were calculated as described by Dunn and Brown [35]. The following parameters were estimated: (i) cell area (μm^2^); (ii) extension, i.e. a measure of how much the shape differs from a circle, taking a value of zero if the shape is circular and increasing without limit as the shape becomes less compact. The extension is the sum of elongation and dispersion; (iii) dispersion, i.e. the minimum extension that can be attained by compressing the shape uniformly. The minimum dispersion of zero is only achieved if the shape is an ellipse and dispersion can never take a value greater than that of extension; (iv) elongation, a measure of how much the shape must be compressed along its long axis to minimize its extension. The elongation can never take a value of less than zero or greater than the extension. The minimum elongation of zero is only achieved if the shape is a circle and increases without limit as the shape becomes more elongated.

### Exposition of Cells to dcEFs

WC 256 cells were exposed to dcEFs at a strength of 1–3 V/cm in the plexiglass apparatus described in detail by Korohoda et al. [13]. Briefly, dcEFs were applied for a specified time, up to 2.5 hours, through Ag/AgCl reversible electrodes of 6 cm^2^ immersed in saline-filled wells connected by agar bridges (2% agar in 0.5 n KCl, 8 cm long) to neighboring wells, to which the observation chambers were attached. The observation chambers were made of cover glasses measuring: 60 x 35 x 0.2 mm. The investigated cells were plated for 2 hrs onto one of the cover glasses at a density of 55 000 (BC) or 35 000 (LC) cells/cm^2^, and incubated in RPMI-1640 supplemented with 5% FCS in a humidified atmosphere with 5% CO_2_ at 37°C. We use different plating densities because BC cells have a smaller surface area and it was possible to plate more cells in the electrotactic chamber without physical contact between cells and analyze more BC cells in one experiment. Then the chamber was mounted with silicone grease in the plexiglass apparatus.

In some experiments WC256 cells were pre-incubated with 50 μM NSC23766 (Rac1 inhibitor), 50 μM ZCL278 (Cdc42 inhibitor III), 30 μM Rhosin (Rho inhibitor), 10 μM Y-27632 (ROCK inhibitor), 50 μM blebbistatin (myosin II inhibitor), 10 μM ML-7 (MLCK inhibitor) for 30 minutes in RPMI-1640 with 5% FCS. To investigate the role of intracellular Ca^2+^ in electrotaxis, cells were loaded with 30 μM BAPTA-AM for 30 minutes in serum-free RPMI-1640.

### Time-Lapse Monitoring of Movement of Individual Cells

The movement of WC256 cells in isotropic conditions and in dcEFs (1V/cm, 2V/cm, 3V/cm) was time-lapse recorded for 30 minutes (BC) or 2.5 hr (LC) at time intervals 15 sec. or 2.5 min., respectively. The tracks of individual cells were determined from the series of changes in cell centroid positions, pooled and analyzed as previously described [11, 36]. The following parameters were estimated: (i) the displacement length (μm), i.e. the distance from the starting point directly to the cell's final position, (ii) the displacement speed (μm/min), i.e. the distance from the starting point directly to the cell's final position/time of recording, (iii) the trajectory speed (μm/min), i.e. trajectory length/time of recording, (iv) the coefficient of movement efficiency (CME) corresponding to the ratio of cell displacement to cell trajectory length, (v) average directional cosines γ; γ is defined as the directional angle between the 0X axis (parallel to the field direction) and the vector AB. A and B are the first and subsequent positions of the cell, respectively [36, 37, 38, 39]. Cell trajectories from no less than three independent experiments (number of cells = 50) were taken for the estimation of statistical significance by the non-parametric Mann-Whitney test: p< 0.05.

### SEM Analysis

The cells were fixed with 2.5% glutaric aldehyde in 0.1 M cacodylic buffer, rinsed with 0.1 M cacodylic buffer several times, subjected to osmium post-fixation for 1 h, and then washed 3 times in 0.1 M cacodylic buffer. The samples were subsequently dehydrated in increasing concentrations of ethanol (50–100%), rinsed with 100% acetone, dried at the critical point (− 200°C for 40 min) and left overnight under vacuum. The dehydrated samples were coated with a thin film of gold (JFC-1100F) and observed by SEM (JEOL JSM-5410) [40].

### Rho Family GTPase Activation Assays

Colorimetric G-LISA activity assay kits were used according to the manufacturer’s instructions to quantitatively assess GTP-bound RhoA, Rac1, and Cdc42 levels in WC256 cells. The investigated cells were plated for 2 hours into the electrotactic chamber at a density of 10^6^ cells/cm^2^, and incubated in RPMI-1640 supplemented with 5% FCS, followed by dcEF stimulation at the strength of 3 V/cm for 5 and 15 minutes. Then cells were washed with ice-cold PBS, lysed in ice-cold lysis buffer and centrifuged at 14000 x g at 4°C for 2 minutes. Supernatants were collected, snap frozen in liquid nitrogen and stored at −80°C until used. GTP-bound RhoA, Rac1, or Cdc42 levels were then determined using the RhoA-GTP, Rac1-GTP, Cdc42-GTP binding 96-well plates, including a lysis buffer blank control and GTP-bound recombinant positive controls. Absorption of the ELISA wells was determined with VersaMax ELISA Microplate Reader (Molecular Devices) at 490 nm.

### p^CMV^ LifeAct-TagGFP2 Plasmid Propagation

p^CMV^ LifeAct-TagGFP2 (Ibidi) plasmid was propagated in DH5alpha *E*. *coli* host strain in LB medium with kanamycin (30 μg/ml), isolated and purified with Plasmid Midi AX (A&A BIOTECHNOLOGY).

### Transfection of Blebbing WC256 Cells

Cultured cells were harvested and resuspended in RPMI-1640 medium and plasmid DNA p^CMV^ LifeAct-TagGFP2 was added to a final concentration of 80 μg/ml. Then cells were kept on ice for 5 minutes, followed by delivery to a Gene Pulser Cuvette (Bio-Rad no 165–2088 with 0.4 cm electrode) and electrical pulses were applied to the cells using a MicroPulser™ Electroporator (BIO-RAD) with the following settings: resistance- ∞, capacitance—950 μF, voltage—250 V. After electroporation, cells were immediately transferred into 5 ml of RPMI-1640 with 5% FCS and antibiotics and then replaced with fresh medium after 24 hours. The entire procedure was carried out at room temperature.

### Transfection of Lamellipodia Forming WC256 Cells

Cells were seeded 24 hours before transfection onto a 24-well plate. Before the addition of lipoplexes, cells were washed twice with Opti-MEM® I medium without FCS and antibiotics. Lipoplexes consisting of 1.7 μl Lipofectamine 2000 Reagent and 1 μg of p^CMV^ LifeAct-TagGFP2 plasmid DNA were added to cells which were then incubated for 7 hours at 37°C. Then RPMI-1640 medium was added to achieve the standard concentration of serum and antibiotics. This mixture was replaced after 24 hours post transfection with fresh RPMI-1640 medium with 5% FCS and antibiotics. We used two different methods to transfect the same construct into the BC and LC sublines because BC cells were difficult to transfect by standard lipofection.

### Proteomic Analysis

1.7 x 10^6^ cells were lysed in 300 μl of 4% SDS and 0.1 M DTT in Tris-HCl pH 7.6 and sonicated for 10 min (320 W, 30 s on/off) using Bioruptor^TM^ UCD-200 (Diagenode). Then samples were incubated at 95°C for 5 min, centrifuged at 30 000 x g for 30 min at 20°C and prepared for Filter Aided Sample Preparation (FASP) for LC-MS/MS analysis based on procedures described by Wisniewski et al. [41]. The peptide content was estimated by UV light spectral density at 280 nm (Nano-Drop, Thermo). *FASP* peptides were analyzed by mass spectrometry (MS) using an UltiMate 3000RS LC nanoSystem (Dionex) coupled with a Q-Exactive mass spectrometer (Thermo Fisher Scientific) with DPV-550 Digital PicoView nanospray source. Detailed instructions for performing FASP and liquid chromatography and tandem mass spectrometry (LC-MS/MS) are described in S1 Text. Database searching of RAW files was performed in Proteome Discoverer 1.4 (Thermo Fisher Scientific). MASCOT 2.4.0 was used for database searching against the SwissProt database with Rodentia taxonomy restriction (release May 2014, 26 248 sequences).

## Results

### Morphological Characteristics of Blebbing and Lamellipodia Forming Sublines of WC 256 Carcinosarcoma Cells

An appropriate model is required to analyse the electrotactic migration of blebbing and lamellipodia forming cells, ideally allowing for a comparison of the reactions of cells of the same origin but representing various strategies of cell movement. In a previous study we characterized two sublines of WC 256 carcinosarcoma cells generated by epigenetic changes. Non-adherent cells (forming blebs) were mostly polarized and migrated in a non-adhesive mode which was very efficient but operated only in a 3D environment when cells were placed between 2 pieces of glass (“chimney” chamber), whereas strongly adherent cells (forming lamellipodia) were spread and migrated in an adhesive mode which was less efficient but worked on 2D substrata [34]. However, this model was not suitable for research on electrotactic movement in our electrotactic chamber because adherent blebbing cells were required for these experiments.

**Fig 1:**
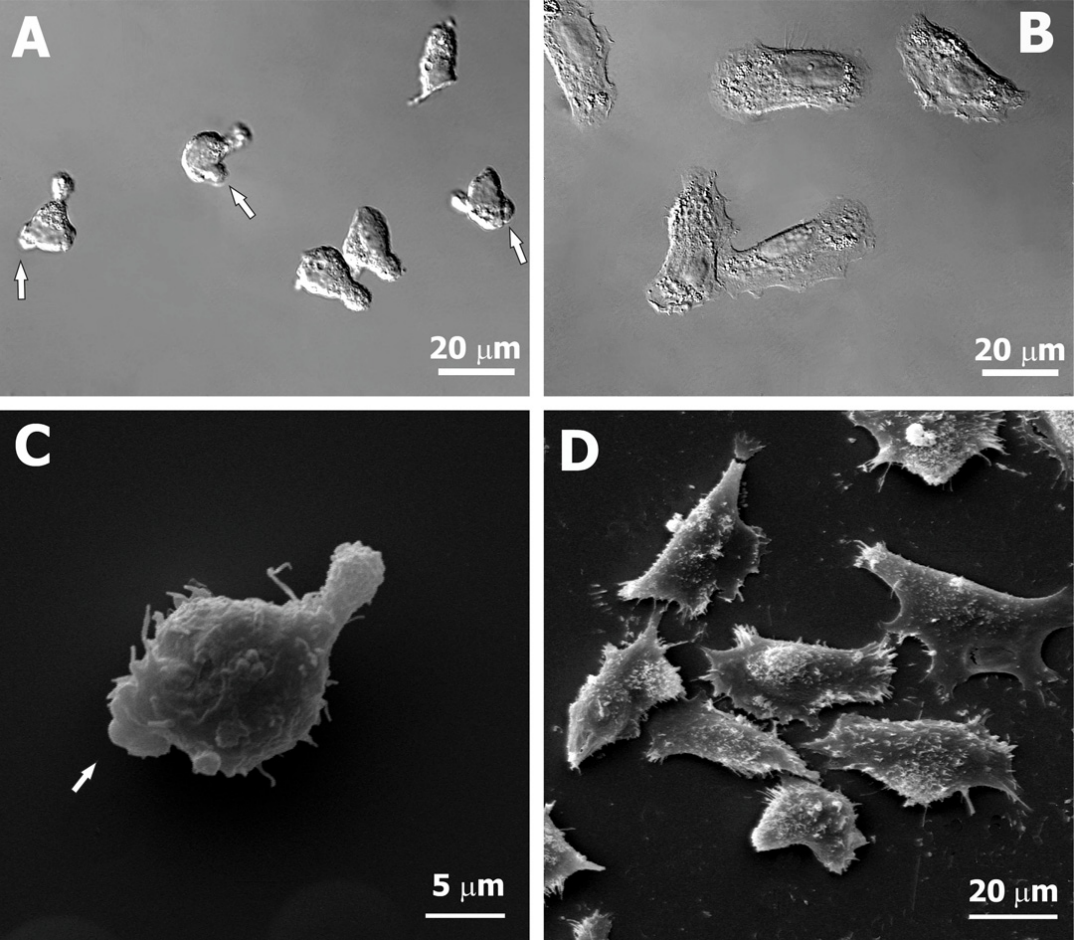
The morphology of two sublines of Walker carcinosarcoma WC 256 cells migrating in an adhesive mode but forming different protrusions. **(A, C)** Weakly adherent cells forming blebs spontaneously (BC); **(B, D)** Strongly adherent cells forming lamellipodia (LC); photographs obtained in DIC optics **(A, B)** or in SEM **(C, D).** Arrows indicate blebs at the leading edges of cells.

To investigate the electrotactic migration of blebbing cells, we selected a weakly adherent, spontaneously blebbing subline of WC 256 cells (BC) by culturing non-adherent cells in the same flask without shaking for 1–2 weeks. The selection resulted in a weakly adherent cell line variant characterized by extensive bleb formation, clearly visible under a light microscope (Fig 1A) and additionally visualized with a scanning electron microscope (Fig 1C).

We constructed a research model well-suited for discerning the mechanisms of electrotaxis of cells representing various modes of cell migration, i.e. two cell sublines adhering to the substrata, moving under the same conditions (2D) but forming different types of protrusions—blebbing cells (subline BC) and previously selected lamellipodia forming cells (subline LC). Apart from formation of diverse types of cell protrusions, the sublines represented different cell morphologies. LC cells had a larger surface area of cell projection and showed significantly decreased elongation in comparison with BC cells (Fig 1B, 1D vs 1A, 1C; Table 1).

**Table 1:** Parameters characterizing the morphology of blebbing and lamellipodia forming cell.

Parameters (±SEM)^#^	BC	LC
Mean area (μm^2^)	419.90 ± 18.40	677.20 ± 29.40*
Mean extension	0.81 ± 0.04	0.71 ± 0.03
Mean dispersion	0.14 ± 0.01	0.15 ± 0.02
Mean elongation	0.71 ± 0.04	0.57 ± 0.04*

^**#**^Definitions of the parameters and details of the statistics are given in Materials and Methods

*Statistically significant BC vs. LC (p<0.05)

**Fig 2:**
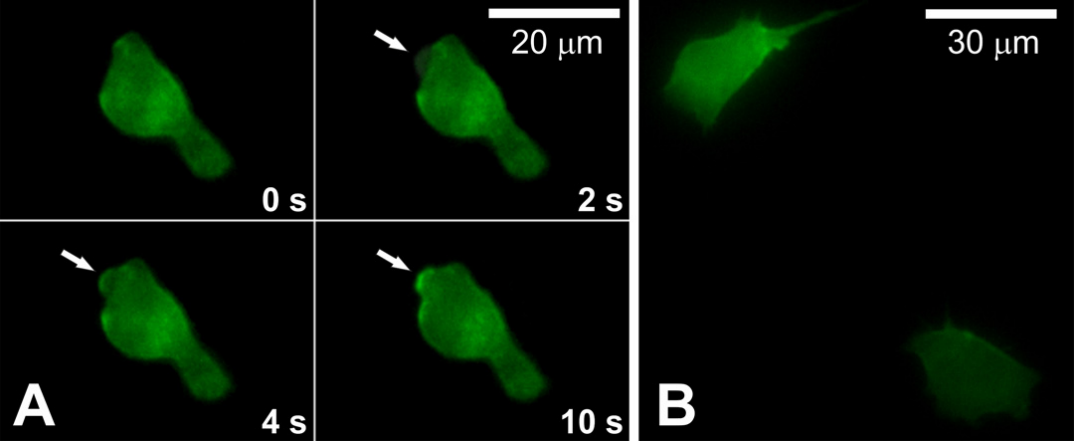
Distribution of F-actin in migrating BC and LC cells. Cells were transfected with LifeAct, a 17-amino-acid peptide, which stains F-actin in eukaryotic cells. (**A**) Sequence of frames showing bleb formation (indicated by arrow) in weakly adherent BC cells. **(B)** Strongly adherent lamellipodia forming LC cell.

To characterise the bleb life cycle in migrating BC cells, we used LifeAct, a 17-amino-acid peptide which stained filamentous actin (F-actin) structures in eukaryotic cells [42] and quantified the motility of transfected cells by time-lapse video microscopy (Fig 2A). In BC cells we observed a characteristic bleb life cycle, representing three phases: bleb initiation, bleb expansion and bleb retraction [3]. However, in migrating cells bleb retraction does not always occur and sometimes the bleb is stabilised and the cell body moves forward. Bleb formation is preceded by the separation of the membrane from the cortex, development of cellular extension (initially free of actin filaments), followed by actin cortex reformation at the bleb membrane (S1 Movie). In contrast, in LC cells active lamellipodia formation and turnover along the perimeters of cells was apparent (Fig 2B, S2 Movie).

### Migratory Characteristics of Blebbing and Lamellipodia Forming Sublines of WC 256 Carcinosarcoma Cells

**Fig 3:**
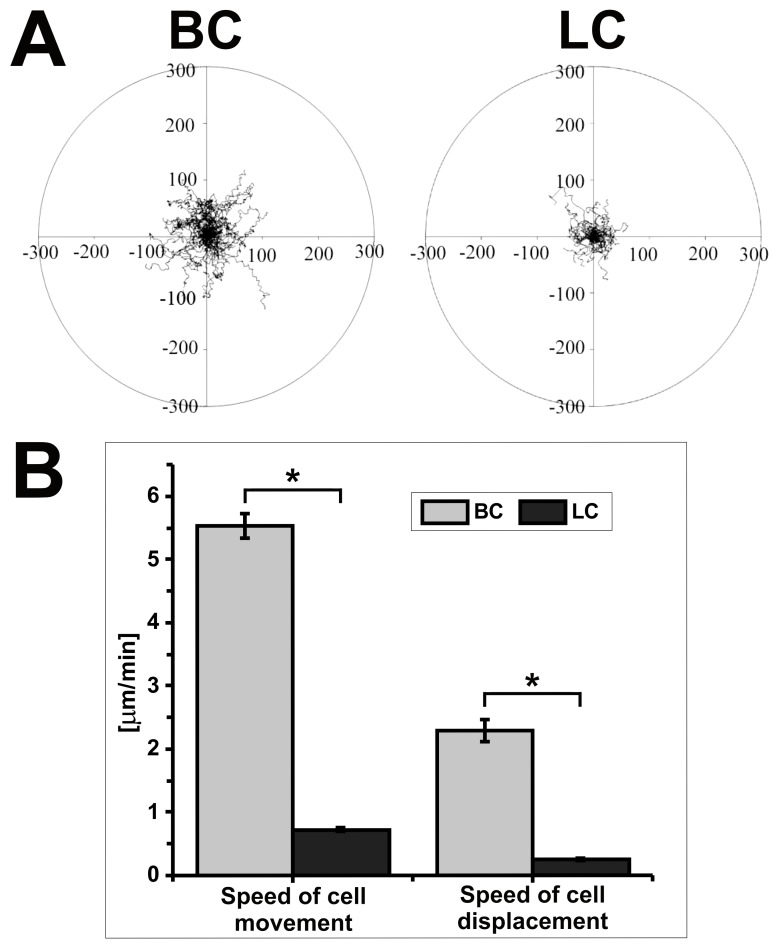
Comparison of motile activity of BC and LC cells. (**A**) Composite trajectories of BC and LC cells migrating under isotropic conditions are shown as circular diagrams. In each diagram, the initial point of each trajectory was placed at the center of the circle. Each trajectory was constructed from 120 (BC) or 48 (LC) successive positions of cell centroids recorded at 15-sec (BC) or 150-sec (LC) time intervals, respectively. The movement of BC and LC was recorded for 30 minutes and 150 minutes, respectively. (**B**) Diagram showing the speed of cell movement and speed of cell displacement (n = 50); *Statistically significant (p<0.05).

Next, we characterized the motile activity of both sublines of WC256 cells forming different protrusions. As shown in Fig 3, Table 2 and Table 3, BC migrated much more efficiently (S3 Movie) than LC cells (S4 Movie). The rate of cell displacement (the distance from the starting point directly to the cell's final position/time of recording) was 9 times higher for BC cells than for LC cells (2.29 ± 0.17 μm/min and 0.25 ± 0.02 μm/min, respectively) and the speed of cell movement (total length of cell trajectory/time of recording) was about 8 times higher (5.53 ± 0.19 μm/min and 0.72 ± 0.03 μm/min, respectively).

**Table 2:** Parameters characterizing BC cell migration under isotropic conditions and after the application of an electric field.

BC				
Parameters (±SEM)^#^	Electric field [V/cm]			
	0	1	2	3
Trajectory length [μm]	165.87 ± 5.79	172.59 ± 10.69	186.33 ± 8.48	200.15 ± 7.37*
Trajectory speed [μm/min]	5.53 ± 0.19	5.75 ± 0.36	6.21 ± 0.28	6.67 ± 0.24*
Displacement length [μm]	68.92 ± 5.16	73.69 ± 6.28	107.24 ± 6.62*	127.44 ± 6.64*
Displacement speed [μm/min]	2.29 ± 0.17	2.45 ± 0.21	3.47 ± 0.22*	4.25 ± 0.22*
Coefficient of movement efficiency CME	0.41 ± 0.09	0.45 ± 0.03	0.59 ± 0.03*	0.64 ± 0.02*
Average directional cosine γ	0.06 ± 0.02	0.14 ± 0.01*	0.54 ± 0.08*	0.78 ± 0.04*

^#^Definitions of the parameters and details of the statistics are given in Materials and Methods

*Statistically significant vs. 0 V/cm (p<0.05)

**Table 3:** Parameters characterizing LC cell migration under isotropic conditions and after the application of an electric field.

LC				
Parameters (±SEM)^#^	Electric field [V/cm]			
	0	1	2	3
Trajectory length [μm]	108.71 ± 4.33	96.37 ± 5.57	147.17 ± 5.54*	166.49 ± 8.39*
Trajectory speed [μm/min]	0.72 ± 0.03	0.64 ± 0.04	0.98 ± 0.04*	1.11 ± 0.05*
Displacement length [μm]	37.62 ± 2.63	35.89 ± 2.43	93.58 ± 4.23*	96.97 ± 3.56*
Displacement speed [μm/min]	0.25 ± 0.02	0.24 ± 0.02	0.62 ± 0.03*	0.65 ± 0.02*
Coefficient of movement efficiency CME	0.34 ± 0.02	0.39 ± 0.02	0.64 ± 0.02*	0.64 ± 0.02*
Average directional cosine γ	0.05 ± 0.01	0.28 ± 0.09*	0.81 ± 0.03*	0.85 ± 0.03*

^#^Definitions of the parameters and details of the statistics are given in Materials and Methods

*Statistically significant vs. 0 V/cm (p<0.05)

### Proteomic Composition of Blebbing and Lamellipodia Forming Sublines of WC 256 Carcinosarcoma Cells

Shotgun LC-MS/MS analysis of protein contents in three biological replicates for each WC 256 subline was performed. Taking into consideration FDR<0.01 and only high confidence peptides, reasonable and very reproducible numbers of proteins were identified in all six studied samples, i.e.: 2818, 2868, 2947 (mean 2878) and 2870, 2933, 3022 (mean 2942) for BC and LC cells, respectively. From the comparison of protein sets obtained for both sublines, we found 54 proteins exclusively identified in BC cells and 77 proteins occurring solely in LC cells, wherein the following criteria were adopted: a given differential protein was identified in at least two of three biological replicates within one group on the basis of two or more unique peptides and no peptide belonging to this protein was identified in the samples of the second group. Differentially expressed proteins are listed in S1 Table. We performed a bioinformatic analysis of two sets of differentially expressed proteins with the DAVID Functional Annotation Tool (http://david.abcc.ncifcrf.gov/summary.jsp, DAVID Bioinformatics Resources 6.7) and comparative Gene Ontology (GO) analysis using the FatiGO Tool (http://babelomics.bioinfo.cipf.es/). We did not find any statistically significant GO group (including those engaged in migration or adhesion processes) which would differentiate the compared sets of proteins, however the analysis was hampered because it included proteins with overall Rodentia taxonomy (S1 Table).

These results suggest that both sublines use a similar molecular machinery for cell movement and the different modes of migration result from differences in the regulation of activity of proteins involved in these processes rather than in differences in protein expression.

### Electrotaxis of Blebbing and Lamellipodia Forming Sublines of WC 256 Carcinosarcoma Cells in an Electric Field

**Fig 4:**
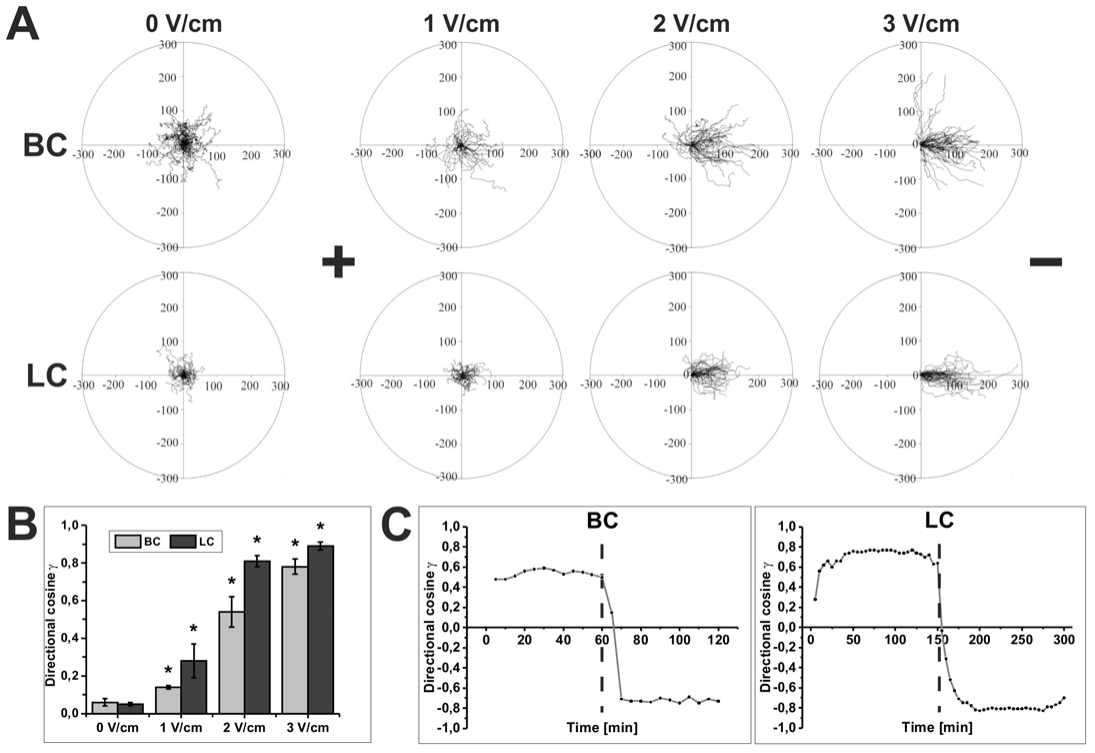
The effect of dcEF on the migration of BC and LC cells. **(A)** Composite trajectories of BC and LC cells migrating in the absence and in the presence of dcEFs are shown as circular diagrams. In each diagram, the initial point of each trajectory was placed at the center of the circle. The x-axis corresponds to the direction of the electric field. The cathode was always placed at the right side of the diagram. Each trajectory was constructed from 120 (BC) or 48 (LC) successive positions of cell centroids recorded at 15-sec (BC) or 150-sec (LC) time intervals, immediately after the exposure of cells to dcEF. The movement of BC and LC was recorded for 30 minutes and 150 minutes, respectively. (**B**) Diagrams presenting the values of directional cosines γ for BC and LC cells depending on applied field strength; mean ± SEM; p<0.05 vs 0 V/cm (**C**) The dynamics of reversibility of the direction of cell movement. The directionality of cell movement was completely reversible upon reversing the field polarity (3V/cm). For both cell sublines the average directional cosines γ were analyzed every 5 minutes. Perpendicular lines indicate the time when the electric field polarity was reversed (n = 50). *Statistically significant vs. 0 V/cm (p<0.05).

As described above, in the absence of any external electric field, BC cells migrated faster and more efficiently than LC cells but both sublines showed random directionality of cell movement (cosine γ = 0.06 ± 0.02 and 0.05 ± 0.01, respectively) (Table 2 and Table 3). A dramatic change occurred in both sublines following the application of an electric field, whereby their movements became strongly directional and cells turned towards the cathode (Fig 4). The effects of the electric field on the motility of BCs were enhanced as the applied voltage gradient was made stronger (Fig 4A). Thus, as the electric field was increased from 1 V/cm to 3 V/cm, both the directionality of movement and the overall length of cell displacement increased steadily (cos γ = 0.14 ± 0.01; 0.54 ± 0.08; 0.78 ± 0.04 for cells moving in dcEF of 1 V/cm; 2 V/cm and 3 V/cm, respectively) (Fig 4B, Table 2). We also observed that the application of dcEFs of 2 and 3 V/cm, but not of 1V/cm, resulted in a significant increase in cell displacement compared to the control (Table 2). A similar reaction to dcEF was observed in the case of LC cells. The increase of the strength of dcEF significantly enhanced cell directionality toward the cathode (cosine γ = 0.28 ± 0.09; 0.81 ± 0.03; 0.85 ± 0.03 for cells moving in dcEF of 1 V/cm; 2 V/cm and 3 V/cm, respectively) and dcEF of 2 V/cm and 3V/cm induced a significant increase of cell displacement (Fig 4B, Table 3). The effects of dcEF on both cell sublines were reversible. To determine the dynamics of the reversibility of the direction of LC and BC cell migration after reversing the field, we analyzed the changes of the values of average directional cosines γ every 5 minutes (i.e. the same time increment for both sublines). As shown in Fig 4 the directionality of cell movement was completely reversible upon reversing the field polarity (3V/cm). Interestingly, full reversal of cell direction (i.e. value of directional cosines γ about– 0.8) after the change of dcEF polarity was much faster in the case of BC (10 minutes) than LC cells (30 minutes). However, the first observed reaction (i.e. decrease in directionality) after reversing field polarity was much faster (less than 5 minutes) and similar in both cell lines. Moreover, differences in the mode of change in direction of movement were found. About 40% of BC and 70% of LC cells reversed by U-shape turning and the remaining cells by repolarization (Fig 4C, S5 Movie, S6 Movie).

### The Effect of Rac, Cdc42 and Rho Inhibitors on Electrotactic Movement of Blebbing and Lamellipodia Forming Sublines of WC 256 Carcinosarcoma Cells

Simplified models of cell migration assume that actin polymerization-based protrusions or blebs driven by actomyosin contractility are fundamental for plasticity in the mode of cell migration [43]. Rho family GTPases are involved in the regulation of actin, contraction and adhesion dynamics that are crucial for cell migration. It is postulated that Rac promotes membrane protrusion at the leading edge by induction of actin polymerisation, Cdc42 is required for filopodium extension and Rho regulates contractility in the cell body [18]. In general, it is postulated that Rho activation supports bleb-based migration and Rac induces actin polymerization-based protrusions. It was also demonstrated that Rho GTPases regulate the directional movement of cells, including electrotaxis [30]. Therefore, we tested the relative requirements for Rac, Cdc42 and Rho and their effectors on electrotactic guidance of blebbing and lamellipodia forming sublines of WC 256 carcinosarcoma cells.

**Fig 5:**
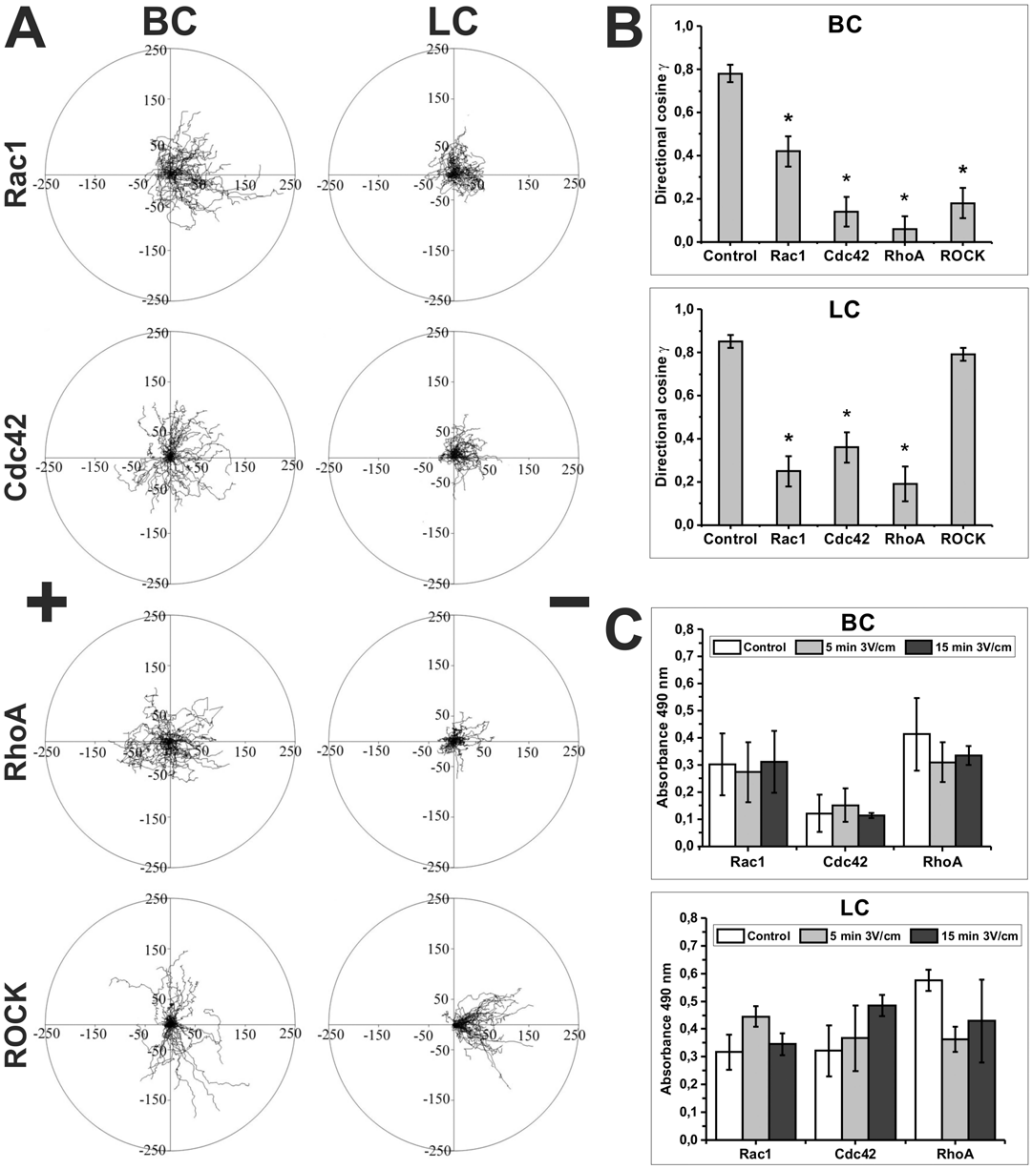
The effect of Rac, Cdc42, Rho and ROCK inhibitors on electrotaxis of BC and LC cells and the effect of dcEF on the activity of small Rho GTP-ases. **(A)** Composite trajectories of BC and LC cells migrating in the presence of dcEF (3 V/cm) and 50 μM NSC23766 (Rac1 inhibitor), 50 μM ZCL278 (Cdc42 inhibitor III), 30 μM Rhosin (Rho inhibitor) or 10 μM Y-27632 (ROCK inhibitor) are shown as circular diagrams. In each diagram, the initial point of each trajectory was placed at the center of the circle. The x-axis corresponds to the direction of the electric field. The cathode was always placed at the right side of the diagram. Each trajectory was constructed from 120 (BC) or 48 (LC) successive positions of cell centroids recorded at 15-sec (BC) or 150-sec (LC) time intervals, immediately after exposure of cells to dcEF. The movement of BC and LC was recorded for 30 minutes and 150 minutes, respectively (n = 50). (**B**) The diagrams depict the values of directional cosines γ. (**C**) The activity of Rac1, Cdc42 and RhoA in BC and LC cells under isotropic condition and after exposition of cells to dcEF for 5 and 15 minutes determined by the G-Lisa assay. *Statistically significant vs. 3 V/cm (p<0.05).

Cdc42 and Rho inhibitors hindered electrotaxis of both BC and LC cells, suggesting that these proteins are necessary for a directional reaction of both sublines in an electric field (3V/cm). Interestingly, the observed effect was stronger in the case of BC cells. Significant differences were shown for Rac1 and ROCK kinase inhibitors in relation to both sublines. As shown in Fig 5A and 5B, Table 4 and Table 5, inhibition of Rac1 impeded directional movement in dcEF of both BC and LC cells, however the effect was much stronger for lamellipodia forming cells (cos γ = 0.42 ± 0.07 for BC and 0.25 ± 0.07 for LC) and the difference was statistically significant (p<0.05). Enormous differences in the reaction of LC and BC cells were observed after ROCK inhibition. It was found that ROCK activity was required for directional movement of BC but not for LC cells (0.18 ± 0.07 and cos γ = 0.79 ± 0.03, respectively). The effects of inhibitors of Rho GTPases on the speed of movement of BC and LC cells under isotropic conditions without the application of an electric field was only moderate or statistically insignificant. Only Rhosin (Rho inhibitor) inhibited the speed of movement of BC cells to 72% of the speed of control cells (S2 Table). These observations suggest that for the sensing of an electric field, the molecular machinery responsible for regulation of actin polymerization may be more relevant for LC than BC cells. On the other hand, proteins directly involved in induction of cell contractility are more relevant for BC cells.

**Table 4:** Quantitative data showing the effects of Rac, Cdc42, Rho and ROCK inhibitors on electrotaxis of BC cells.

BC					
Parameters (±SEM)^#^	Control	Rac1 Inhibitor (NSC23766)	Cdc42 Inhibitor (ZCL278)	Rho Inhibitor (Rhosin)	ROCK Inhibitor (Y-27632)
	3 V/cm				
Trajectory length [μm]	200.15 ± 7.37	192.46 ± 8.30	166.50 ± 7.59*	190.02 ± 9.34*	154.33 ± 8.20*
Trajectory speed [μm/min]	6.67 ± 0.24	6.52 ± 9.28	1.42 ± 0.06*	6.33 ± 0.31*	5.23 ± 0.28*
Displacement length [μm]	127.44 ± 6.64	86.84 ± 5.91*	71.59 ± 5.24*	62.13 ± 4.65*	60.54 ± 8.11*
Displacement speed [μm/min]	4.25 ± 0.22	2.89 ± 0.20*	2.39 ± 0.17*	2.07 ± 0.15*	2.02 ± 0.27*
Coefficient of movement efficiency CME	0.64 ± 0.02	0.47 ± 0.03*	0.43 ± 0.03*	0.34 ± 0.02*	0.34 ± 0.04*
Average directional cosine γ	0.78 ± 0.04	0.42 ± 0.07*	0.14 ± 0.07*	0.06 ± 0.07*	0.18 ± 0.07*

^**#**^Definitions of parameters and details of the statistics are given in Materials and Methods

*Statistically significant vs. 3 V/cm (p<0.05)

**Table 5:** Quantitative data showing the effects of Rac, Cdc42, Rho and ROCK inhibitors on electrotaxis of LC cells.

LC					
Parameters (±SEM)^#^	Control	Rac1 Inhibitor (NSC23766)	Cdc42 Inhibitor (ZCL278)	Rho Inhibitor (Rhosin)	ROCK Inhibitor (Y-27632)
	3 V/cm				
Trajectory length [μm]	166.49 ± 8.39	131.95 ± 4.40*	108.59 ± 5.08*	83.93 ± 4.17*	140.81 ± 6.64*
Trajectory speed [μm/min]	1.11 ± 0.05	0.88 ± 0.03*	0.72 ± 0.03*	0.56 ± 0.03*	0.94 ± 0.04*
Displacement length [μm]	96.97 ± 3.56	42.80 ± 3.23*	44.42 ± 4.22*	26.46 ± 2.89*	76.23 ± 5.84*
Displacement speed [μm/min]	0.64 ± 0.02	0.28 ± 0.02*	0.29 ± 0.03*	0.18 ± 0.02*	0.51 ± 0.04*
Coefficient of movement efficiency CME	0.64 ± 0.02	0.32 ± 0.02*	0.29 ± 0.03*	0.31 ± 0.03*	0.52 ± 0.02*
Average directional cosine γ	0.85 ± 0.03	0.25 ± 0.07*	0.36 ± 0.07*	0.19 ± 0.08*	0.79 ± 0.03

^**#**^Definitions of parameters and details of the statistics are given in Materials and Methods

*Statistically significant vs. 3 V/cm (p<0.05)

Since the first reactions of cells to dcEFs were observed in less than 5 minutes (Fig 4C), in subsequent experiments we used G-LISA assays to test if stimulation of cells with dcEF for 5 and 15 minutes activates Rac, Cdc42 or Rho proteins. However, we did not observe any significant differences as compared to the control for all examined GTP-ases and both sublines (Fig 5C). This result suggests that the application of dcEFs is rather not involved in direct bulk activation of Rho-GTPases in electrotactic cells. The postulated role of these proteins in regulation of cell electrotaxis may be due to the specific localization of activated G-proteins in cells migrating in dcEFs.

### The Effects of Calcium Ions on Electrotactic Migration of Blebbing and Lamellipodia Forming Sublines of WC 256 Carcinosarcoma Cells

**Fig 6:**
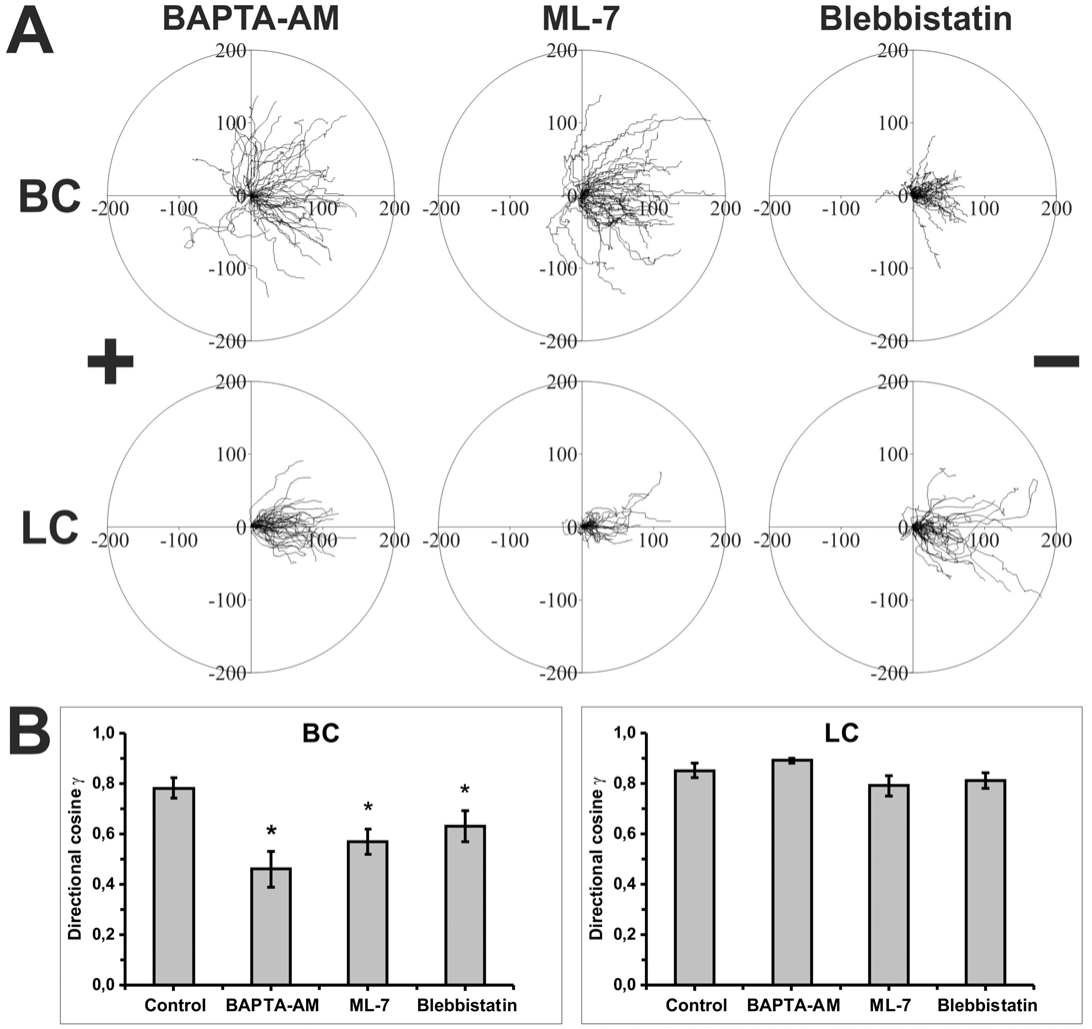
The role of intracellular Ca^2+^ and myosin II in electrotaxis of BC and LC cells. **(A)** Composite trajectories of BC and LC cells migrating in the presence of dcEF (3 V/cm) and 30 μM BAPTA-AM (chelator of [Ca^**2+**^]_i_), 10 μM ML-7 (MLCK inhibitor) or 50 μM blebbistatin (myosin II inhibitor) shown as circular diagrams. In each diagram, the initial point of each trajectory was placed at the center of the circle. The x-axis corresponds to the direction of the electric field. The cathode was always placed at the right side of the diagram. Each trajectory was constructed from 120 (BC) or 48 (LC) successive positions of cell centroids recorded at 15-sec (BC) or 150-sec (LC) time intervals, immediately after exposure of cells to dcEF. The movement of BC and LC cells was recorded for 30 minutes and 150 minutes, respectively (n = 50). (**B**) The diagrams depict the values of directional cosines γ. *Statistically significant vs. 3 V/cm (p<0.05).

Regulation of cell migration by the Rho family of small GTPases is a generally accepted concept in which Cdc42 and Rac promote actin polymerization while Rho is responsible for trailing edge retraction and contractility of the cell cortex. We demonstrated that only the electrotactic reaction of BC cells depends on the activity of Rho kinase (ROCK), which activates non-muscle myosin II and induces cell contractility. The results suggest that electrotactic directional movement of blebbing cells is connected to the regulation of the contraction mechanism. However, apart from ROCK, myosin II may also be activated by myosin light chain kinase (MLCK), the activity of which depends on changes in intracellular calcium concentration. Calcium signalling has been proposed to play an important role in the regulation of electrotaxis [32]. Therefore, we tested the effect of the omission of calcium ions on the electrotactic reaction of BC and LC cells. We found that the binding of intracellular calcium by BAPTA-AM reduced the directionality of BC cells by about 43% (directional cosine γ = 0.46 ±0.07) but did not affect the electrotaxis of LC cells (directional cosine γ = 0.89 ± 0.01) (Fig 6, Table 6 and Table 7). The directional reaction of BC cells was also reduced by EGTA, confirming the importance of extracellular Ca^2+^ for electrotactic migration of these cells (data not shown).

**Table 6:** Quantitative data showing the effects of BAPTA, blebbistatin and ML-7 on the electrotaxis of BC cells.

BC				
Parameters (±SEM)^#^	Control	BAPTA-AM	ML-7	Blebbistatin
	3 V/cm			
Trajectory length [μm]	200.15 ± 7.37	151.76 ± 7.22*	170.67 ± 6.12*	82.72 ± 3.57*
Trajectory speed [μm/min]	6.67 ± 0.24	5.05 ± 0.24*	5.68 ± 0.20*	2.75 ± 0.11*
Displacement length [μm]	127.44 ± 6.64	96.56 ± 5.25*	99.88 ± 5.51*	43.43 ± 3.54*
Displacement speed [μm/min]	2.29 ± 0.22	3.21 ± 0.17*	3.33 ± 0.18*	1.45 ± 0.12*
Coefficient of movement efficiency CME	0.64 ± 0.02	0.64 ± 0.02	0.58 ± 0.02	0.50 ± 0.03*
Average directional cosine γ	0.78 ± 0.04	0.46 ± 0.07*	0.57 ± 0.06*	0.63 ± 0.06*

^**#**^Definitions of the parameters and details of the statistics are given in Materials and Methods.

*Statistically significant vs. 3V/cm (p<0.05)

**Table 7:** Quantitative data showing the effects of BAPTA, blebbistatin and ML-7 on the electrotaxis of LC cells.

LC				
Parameters (±SEM)^#^	Control	BAPTA-AM	ML-7	Blebbistatin
	3 V/cm			
Trajectory length [μm]	166.49 ± 8.39	96.19 ± 4.19*	53.05 ± 0.03*	101.92 ± 7.82*
Trajectory speed [μm/min]	1.11 ± 0.05	0.64 ± 0.02*	0.35 ± 0.03*	0.68 ± 0.05*
Displacement length [μm]	96.97 ± 3.56	76.44 ± 4.17*	33.29 ± 4.00*	65.79 ± 6.53*
Displacement speed [μm/min]	0.25 ± 0.02	0.51 ± 0.03	0.22 ± 0.03	0.44 ± 0.04
Coefficient of movement efficiency CME	0.64 ± 0.02	0.77 ± 0.01*	0.59 ± 0.03	0.61 ± 0.03
Average directional cosine γ	0.85 ± 0.03	0.89 ± 0.01	0.77 ± 0.03	0.76 ± 0.03

^**#**^Definitions of the parameters and details of the statistics are given in Materials and Methods.

*Statistically significant vs. 3V/cm (p<0.05)

To confirm the significance of calcium ions in the contraction and electrotactic reaction of BC cells, we analyzed the effect of inhibition of MLCK (by ML-7) and myosin II (by blebbistatin). Both ML-7 and blebbistatin reduced the directionality of BC by about 20% as compared to the control (directional cosine γ = 0.57 ± 0.06 and 0.63 ± 0.06, respectively; 0.78 ± 0.04 for control) but did not affect the electrotaxis of LC (directional cosine γ = 0.77 ± 0.03 and 0.76 ± 0.03 respectively; 0.85 ± 0.03 for control) (Fig 6, Table 6 and Table 7).

The results showed that cell contraction regulated by calcium ions is essential for the electrotactic reaction of blebbing cells but not lamellipodial cells.

## Discussion

The aim of this study was to establish if cells representing different strategies of migration show a similar electrotactic reaction to external electric fields. For this purpose we constructed a unique experimental model–two sublines of Walker carcinosarcoma WC256 cells representing: (i) mesenchymal migration, characterized by strong adhesion and formation of lamellipodia (LC) and (ii) ameboid migration, characterized by weak adhesion and formation of blebs (BC). As both cell sublines were adherent, they were suitable for use in 2D electrotactic chambers [44]. Bleb formation in BC cells was clearly visible under light and scanning electron microscope. Moreover the experiment with LifeAct, which stained F-actin, revealed a classical bleb life cycle in BC cells [3]. Both BC and LC sublines showed efficient cathodal electrotaxis in physiological electric fields and exhibited a dose-dependent response in directional bias to applied electric fields with significant reaction at 1 V/cm and a fully saturated response at 3 V/cm. Analysis of proteomic composition of blebbing and lamellipodia forming cells revealed only slight differences in protein composition, including only single proteins directly engaged in migration or adhesion processes. We therefore hypothesise that BC and LC cells use the same protein set for cell movement and their different migration modes result from differences in protein regulation rather than differences in expression. However, although LC-MS/MS analysis revealed only minor differences in proteomes of both sublines (54 proteins exclusively identified in BC cells and 77 in LC cells per ~3000 proteins) we cannot exclude that some of these proteins are responsible for the different strategies of cell motility of both sublines. In particular, some of these proteins are involved in the regulation of cell migration. For example, LC cells show unique expression of Rho activating protein 18 and formin. Rho GTPase activating protein 18 regulates F-actin polymerization by inhibiting Rho. Its overexpression suppresses the activity of RhoA, disrupts stress fiber formation and is involved in regulation of cell shape, spreading, and migration [45]. On the other hand, formins are involved in the regulation of actin polymerization and regulate cell migration [46]. However, only further research may clarify the significance of these differences in protein expression for both lamellipodial types of migration and electrotactic reaction. An interesting screening strategy for the discovery of proteins important in galvanotaxis was recently proposed by Nakajima et al. [47]. This strategy involved a large scale RNAi method for electrotaxis phenotype, allowing to simultaneously screen large numbers of different cells transfected with different siRNAs. This method may simplify further research on the significance of differences in protein expression in LC and BC cells for electrotactic migration. Electrotactic migration was reported for several cell types representing different strategies of movement [23, 24, 25, 26], however to our knowledge this is the first report demonstrating that sublines of the same cell type representing blebbing or lamellipodial migration show the same electrotactic reaction. Our observations strongly suggest that blebbing, similarly to lamellipodia formation, enables directed cell motility in external electric fields.

Currently, a significant role of blebbing in cell migration is generally accepted and the mechanism of bleb formation is well characterized [3], however knowledge on extracellular signals that initiate the formation of blebs at the front of migrating cells and subsequent directional movement is limited. Nevertheless, it was reported that the location of blebs may be controlled directly by a gradient of chemoattractants. For instance, Yoshida and Soldati demonstrated that in *Dictyostelium discoideum* bleb formation is under the control of chemotactic signalling and cAMP induces bleb formation at the leading edge of cells migrating toward the cAMP source [7]. On the other hand, Blaser et al. [6] reported biased bleb formation in zebrafish primordial germ cells in response to a SDF-1 gradient. Directional migration resulted from a local increase in Ca^+2^ concentration after CXCR4 receptor activation and actomyosin contraction at the leading edge of migrating cells. Our observations suggest that similarly to chemotactic factors, electric fields may also induce biased formation of various cell protrusions (i.e. blebs and lamellipodia) to prompt directional movement. For instance, the asymmetric formation of filopodium growth induced by electric fields was observed in human lung adenocarcinoma cells CL1-5 and CL1-0 [48].

The results presented by Blaser et al. [6] and Yoshida and Soldati [7] revealed that the same or similar chemotactic pathways and factors may be involved in induction of chemotactic migration driven by blebs or lamellipodia. One of the leading models explaining the mechanisms of electrotaxis follows the chemotaxis paradigm. It was proposed that in cathodal electrotaxis asymmetric signalling results from an increase in the number of receptors for chemoattractants in an electric field on the cathode-facing side of the cell. In consequence, the intracellular signal induced by activated receptors is higher at the cathode-facing side of the cell, which becomes the leading edge and the cell moves toward the cathode [18]. Assuming that activated receptors may induce formation of both lamellipodia or/and blebs, this may explain why both sublines, i.e. LC and BC cells, effectively react to electric fields migrating towards the cathode. Altogether, this suggests that during electrotactic migration both types of protrusions may be efficiently utilized for directional migration. This is consistent with observations that blebs and lamellipodia cooperate during chemotaxis and that rapid transitions between various types of protrusions is an imminent feature of cell migration [4, 49].

Although the electrotactic reactions of BC and LC cells were similar, some significant differences were also observed. Upon reversing the field, in both sublines the cells' corresponding responses were rapidly reversed, however this reaction was much faster in BC cells. Full reversal of movement was observed in BC cells after 10 min and in LC cells after 30 min after reversing the field polarity (directional cosine ~ -0.8). However, it should be noted that the directional response of cells was abolished (directional cosine ~0) in five minutes after field reversal in both BC and LC cells (Fig 4). Moreover, in both cell sublines the electric field produced observable morphological and directional changes in the cells within 30 seconds after field reversion (data not shown). This may suggest that the dynamics of the WC 256 cell reaction for field reversion is similar for both sublines but LC cells require a longer time for full reversal of direction of movement simply because these cells migrate more slowly. Therefore, it is possible that the various cell types may use the same sensor(s) for the sensing of electric fields and the same proteins as the molecular machinery for cell movement but because they represent different strategies of migration (for instance blebbing vs. lamellipodial migration) the speed of reaction (i.e. full reversal of movement) may be different, although the dynamics of the primary reaction to the electric field is similar. Moreover, these results are consistent with earlier observations that the first reactions of cells to an electric field are very fast [12, 13].

Since there is a fundamental difference in the mechanisms of bleb and lamellipodia formation, we investigated the potential disparity in the role of Rho GTPases and their effectors for regulation of cell motility in electrotactic migration of blebbing and lamellipodia forming cells. Our results show that for efficient electrotaxis, there is a significant difference in the requirements of BC and LC cells for activation of Rac1 and ROCK proteins. The inhibition of Rac1 GTPase impeded directional movement of LC cells much more strongly than BC cells. On the other hand, the inhibition of ROCK kinase (effector of Rho GTPase) did not affect the directional reaction of LC cells but almost completely abolished the directional reaction of BC cells. However, the activity of Rho GTPases was necessary for the directional reaction of both BC and LC cells in an electric field. Although ROCK is a downstream effector of Rho, in LC cells inhibition of ROCK had no effect on their electrotaxis. Nevertheless, Rho GTPases regulate many effector proteins and affect several cellular events including cytoskeletal organization. For example RhoA activates formins such as mDia proteins at the leading edge and potentially may be involved in actin polymerization in lamellipodia of LC cells [46]. Interestingly, the activity of both Cdc42 and Rho GTPases was necessary for directional reaction of both BC and LC cells in an electric field. A number of studies have described the role of Rho GTPases in cell migration, including electrotaxis [30, 50, 51]. Therefore, our results are unsurprising. However, we show that the electric field as such is unable to activate Rho proteins (Fig 5). Thus it seems unlikely that GTPases are responsible for electrotactic reaction, i.e. they are part of the sensor of an electric field. Nevertheless, it is interesting that for a proper directional reaction the components of the molecular machinery responsible for cell contraction are more important for BC cells and those responsible for actin polymerisation and lamellipodium protrusion are more essential for LC cells. This is consistent with described differences in the molecular mechanisms of protrusion formation in blebbing and lamellipodia forming cells, since blebs are protrusions which expand by hydrostatic pressure generated as a result of actomyosin cortex contraction (promoted by Rho) and lamellipodia are driven by actin polymerisation (promoted by Rac) [9, 46]. Altogether, this suggests that all components of the cellular machinery responsible for cell migration are important for efficient directionality in an electric field, however, depending on the mode of movement, some of these components are more significant than others.

As contractility is not only regulated by the Rho pathway but also by changes in intracellular Ca^2+^ concentration [31], we also tested the effect of calcium ions on electrotactic movement of BC cells. Moreover several reports supported the necessity of extracellular calcium in electrotaxis [32]. Interestingly, we noted a difference in calcium requirement for directional movement of BC and LC cells. In earlier studies it was found that after application of dcEFs, a considerable increase of intracellular calcium was induced in mouse embryo fibroblasts [52], breast cancer cells [53] and a rat osteoblast-like cell line [54], and a high level was maintained as long as cells were exposed to the electric field. In our study we found that the intracellular calcium chelator BAPTA-AM significantly reduced the directionality of BC cells but did not affect electrotaxis of LC cells. The directional reaction of BC cells was also reduced by EGTA, confirming the importance of extracellular Ca^2+^ for electrotactic migration of these cells (data not shown). These results confirm that the molecular mechanisms responsible for cell contraction are more important in electrotaxis of blebbing than lamellipodia forming cells.

The results of our experiments in which we inhibited MLCK or myosin II confirmed the important role of calcium ions in electrotaxis of BC cells. We established that blebbistatin and ML-7 significantly reduced the directionality of BC migration in a dcEF. It is well documented that high acto-myosin tension in blebbing cells is maintained through the actin cortex mostly owing to increasing myosin II activity at the rear part of the cell [55]. Although it was reported previously that keratocyte fragments need myosin II for electrotaxis, we have demonstrated for the first time that myosin II is important for the reaction of blebbing but not lamellipodial cells to dcEF [56].

## Conclusions

In the present study we developed a unique experimental model which allowed for the investigation of electrotactic movement of cells of the same origin but representing different modes of cell migration: weakly adherent, spontaneously blebbing (BC) and lamellipodia forming (LC) WC256 cells. Both blebbing and lamellipodia forming cells exhibit robust cathodal electrotaxis in a physiological EF. However, we demonstrated a significant difference in the requirements of BC and LC cells for an efficient electrotactic reaction. The directional movement of BC cells in EFs depends on proper activity of the molecular machinery responsible for cell contraction (Rho, ROCK, MLCK, myosin II, Ca^2+^). On the other hand, the mechanisms responsible for actin polymerisation and lamellipodium protrusion are more essential for LC cells (electrotactic reaction of LC cells depends on Rac 1 but not on ROCK, MLCK and myosin II activity). In conclusion, our results revealed that both lamellipodia and membrane blebs can efficiently drive electrotactic migration of WC 256 carcinosarcoma cells, however directional migration is mediated by different signalling pathways (S1 Fig).

## Supporting Information

S1 Fig The schematic description of the difference in response of BC and LC cells to EFs during electrotaxis. In BC directional movement in EFs depends mostly on the activity of cell compounds responsible for the generation of cell contraction (Rho, ROCK, MLCK, myosin II, Ca^2+^). In LC, electrotaxis mainly depends on activation of mechanisms responsible for actin polymerisation (Rac, Cdc42, Rho), but not cell contraction.
(TIF)

S1 Movie F-actin dynamics during BC cell migration–stages of bleb formation. Time-lapse imaging of cells expressing LifeAct peptide staining F-actin was performed for 15 min at 2 second intervals. Time acceleration at 60×. Stages of representative bleb formation are shown between 12 min 8 sec and 12 min 18 sec. The region of interest is marked with a frame. Images were obtained with a Leica DMI6000B microscope, equipped with 20× objective, Leica EL6000 metal-halide external light source, GFP filter cube and Leica DFC360 FX CCD camera (the same system was used for S3 Movie). (H.264 MP4; 30 fps; 8,23 MB).
(MP4)

S2 Movie F-actin dynamics during LC cell migration. Time-lapse imaging of cells expressing LifeAct peptide staining F-actin was performed for 30 min at 2 second intervals. Time acceleration at 180×. (H.264 MP4; 90 fps; 8,97 MB).
(MP4)

S3 Movie Motile activity of blebbing WC256 cells (BC). Time-lapse imaging performed for 30 minutes at 15 second intervals. Time acceleration at 300×. Images were obtained with a Leica DM IL LED microscope equipped with 20× objective, Integrated Hoffman Modulation Contrast (IMC) and Moticam 3 CMOS camera (the same system was used for Movies S5, S7 and S8). (H.264 MP4; 20 fps; 9,09 MB).
(MP4)

S4 Movie Motile activity of lamellipodia forming WC256 cells (LC). Time-lapse imaging performed for 150 minutes at 150 second intervals. Time acceleration at 1500×. (H.264 MP4; 10 fps; 7,34 MB).
(MP4)

S5 Movie Electrotaxis and reversibility of direction of BC cell movement after the reversal of electric field polarity. Time-lapse imaging performed for 90 minutes at 15 second intervals. Time acceleration at 300×. Cells were exposed to a dcEF of 3V/cm, 30 minutes with the cathode placed at the right side of the field of view and a further 60 minutes after the reversal of electric field polarity. (H.264 MP4; 20 fps; 9,15 MB).
(MP4)

S6 Movie Electrotaxis and reversibility of direction of LC cell movement after the reversal of electric field polarity. Time-lapse imaging performed for 300 minutes at 150 second intervals. Time acceleration at 1500×. Cells were exposed to a dcEF of 3V/cm, 150 minutes with the cathode placed at the right side of the field of view and a further 150 minutes after the reversal of electric field polarity. (H.264 MP4; 10 fps; 9,41 MB).
(MP4)

S1 Table Differentially expressed proteins in BC and LC cells. (PDF)

S2 Table Quantitative data showing the effects of Rac, Cdc42, Rho and ROCK inhibitors on motile activity of BC and LC cells under isotropic conditions. (PDF)

S1 Text Detailed instructions for *FASP* peptides, liquid chromatography and tandem mass spectrometry. (DOCX)
