# Supplementary figures and images for: Lamellipodia and Membrane Blebs Drive Efficient Electrotactic Migration of Rat Walker Carcinosarcoma Cells WC 256
Source: PLoS One. 2016 Feb 10;11(2):e0149133. doi: 10.1371/journal.pone.0149133 (PMC4749172; doi:10.1371/journal.pone.0149133)

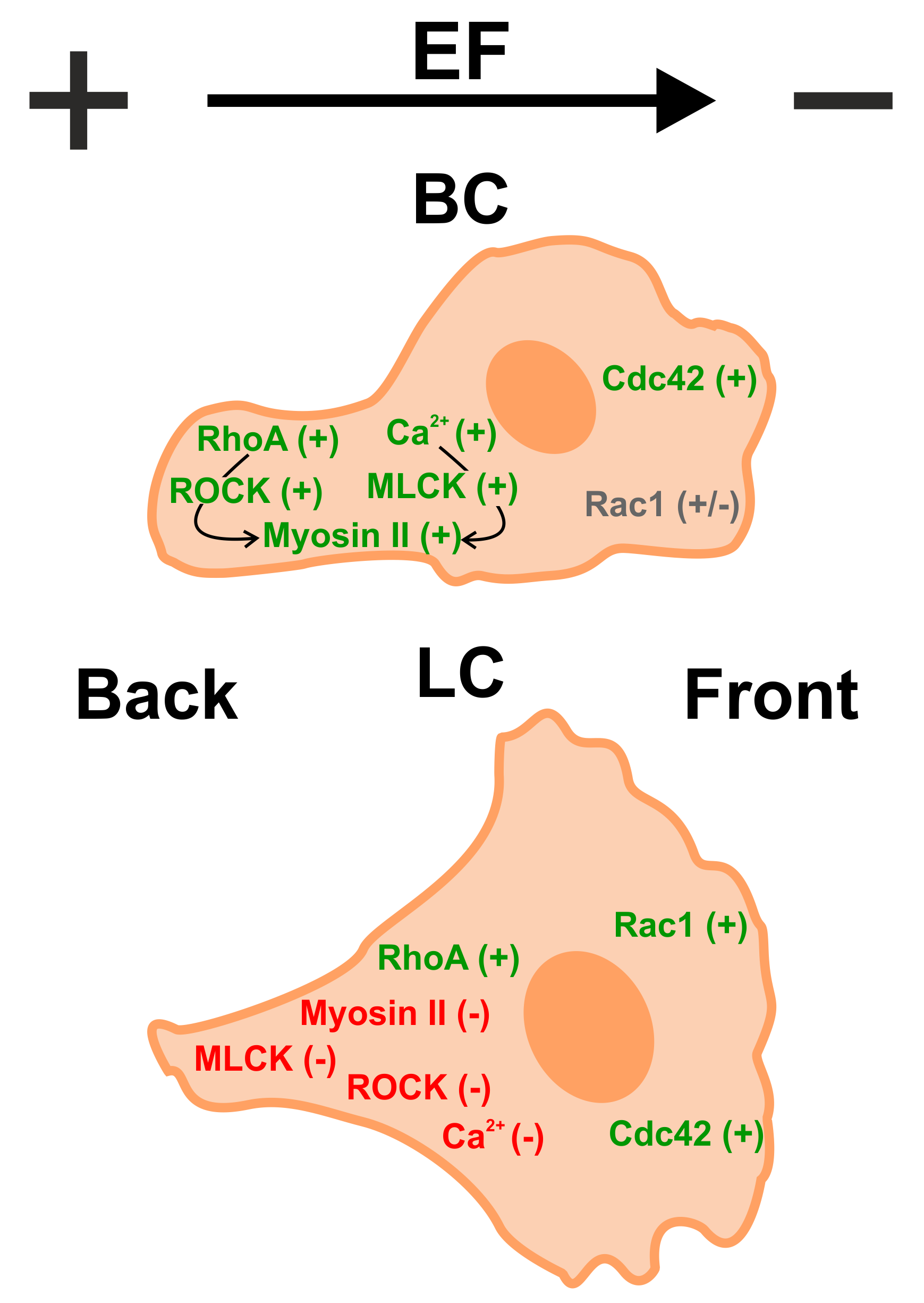

Supplement: S1 Fig — In BC directional movement in EFs depends mostly on the activity of cell compounds responsible for the generation of cell contraction (Rho, ROCK, MLCK, myosin II, Ca2+). In LC, electrotaxis mainly depends on activation of mechanisms responsible for actin polymerisation (Rac, Cdc42, Rho), but not cell contraction. (TIF) [file pone.0149133.s001.tif]
