# Supplementary material for: Lamellipodia and Membrane Blebs Drive Efficient Electrotactic Migration of Rat Walker Carcinosarcoma Cells WC 256
Source: PLoS One. 2016 Feb 10;11(2):e0149133. doi: 10.1371/journal.pone.0149133 (PMC4749172; doi:10.1371/journal.pone.0149133)
Supplement: S1 Table — (PDF) [file pone.0149133.s008.pdf]

Proteins uniquely identified in BC cells	Gene Name	Accession
39S ribosomal protein L13, mitochondrial	Q9D1P0	RM13_MOUSE
60S ribosomal protein L36a	P83882	RL36A_MOUSE
AMME syndrome candidate gene 1 protein homolog	Q9JHT5	AMMR1_MOUSE
Annexin A1	P07150	ANXA1_RAT
ATPase WRNIP1	Q8CG07	WRIP1_RAT
Aurora kinase B	O55099	AURKB_RAT
Beta-adrenergic receptor kinase 1	P26817	ARBK1_RAT
Brain acid soluble protein 1	Q05175	BASP1_RAT
Carboxypeptidase E	P15087	CBPE_RAT
CD2-associated protein	F1LRS8	CD2AP_RAT
Complement component C1q receptor	Q9ET61	C1QR1_RAT
Cytosolic non-specific dipeptidase	Q6Q0N1	CNDP2_RAT
Deubiquitinating protein VCIP135	Q8CF97	VCIP1_RAT
Differentially expressed in FDCP 6	Q8C2K1	DEFI6_MOUSE
Dipeptidyl peptidase 9	Q8BVG4	DPP9_MOUSE
Ferrochelatase, mitochondrial	P22315	HEMH_MOUSE
Frizzled-7	Q61090	FZD7_MOUSE
Galectin-1	P11762	LEG1_RAT
Galectin-8	Q62665	LEG8_RAT
General transcription factor IIE subunit 2	Q9D902	T2EB_MOUSE
Glycerophosphodiester phosphodiesterase domain-containing protein 1	Q0VGK4	GDPD1_RAT
Glycogen phosphorylase, brain form	Q8CI94	PYGB_MOUSE
Heat shock 70 kDa protein 13	O35162	HSP13_RAT
Interferon-inducible double stranded RNA-dependent protein kinase activator A	Q4V8C7	PRKRA_RAT
Lysosomal-associated transmembrane protein 4A	Q60961	LAP4A_MOUSE
Neuropilin-2	O35276	NRP2_RAT
Nuclear fragile X mental retardation-interacting protein 1	Q641W3	NUFP1_RAT
Origin recognition complex subunit 2	Q60862	ORC2_MOUSE
Oxidoreductase HTATIP2	Q9Z2G9	HTAI2_MOUSE
Partner of Y14 and mago	Q8CHP5	WIBG_MOUSE
Pericentriolar material 1 protein	Q9R0L6	PCM1_MOUSE
Peroxiredoxin-2	P35704	PRDX2_RAT
Phosphatidylinositol 4-kinase type 2-alpha	Q99M64	P4K2A_RAT
Proline-, glutamic acid- and leucine-rich protein 1	Q56B11	PELP1_RAT
Proteasome activator complex subunit 4	Q5SSW2	PSME4_MOUSE
Protein arginine N-methyltransferase 7	Q5U4E8	ANM7_RAT
Protein THEMIS	Q8BGW0	THMS1_MOUSE
Protein TSSC4	Q5XIB1	TSSC4_RAT
Purkinje cell protein 4	P63054	PCP4_MOUSE
Raftlin	Q6A0D4	RFTN1_MOUSE
Regulation of nuclear pre-mRNA domain-containing protein 1A	Q8VDS4	RPR1A_MOUSE
rRNA-processing protein UTP23 homolog	Q9CX11	UTP23_MOUSE
Septin-6	Q9R1T4	SEPT6_MOUSE
Serine/threonine-protein kinase DCLK1	O08875	DCLK1_RAT
Signaling threshold-regulating transmembrane adapter 1	Q5M869	SIT1_RAT
Sorting nexin-15	Q4V896	SNX15_RAT
Spartin	Q8R1X6	SPG20_MOUSE
Synaptotagmin-like protein 1	Q99N80	SYTL1_MOUSE
Testis-expressed protein 101	Q924B5	TX101_RAT
TSC22 domain family protein 4	Q3B8N7	T22D4_RAT
Tubulin-specific chaperone cofactor E-like protein	Q5PQJ7	TBCEL_RAT
U1 small nuclear ribonucleoprotein A	Q62189	SNRPA_MOUSE
Vesicle transport through interaction with t-SNAREs homolog 1B	P58200	VTI1B_RAT
Zinc finger MIZ domain-containing protein 2	Q8CIE2	ZMIZ2_MOUSE

Proteins uniquely identified in LC cells	Gene Name	Accession
Acyl-coenzyme A thioesterase 9, mitochondrial	Q9R0X4	ACOT9_MOUSE
ADP-ribosyl cyclase 1	Q64244	CD38_RAT
ADP-ribosylation factor GTPase-activating protein 3	Q9D8S3	ARFG3_MOUSE
AFG3-like protein 2	Q8JZQ2	AFG32_MOUSE
Alpha-1,6-mannosylglycoprotein 6-beta-N-acetylglucosaminyltransferase A	P97259	MGT5A_CRIGR
Alpha-2-macroglobulin receptor-associated protein	Q99068	AMRP_RAT
Armadillo repeat-containing protein 10	B1WBW4	ARM10_RAT
Aspartyl/asparaginyl beta-hydroxylase	Q8BSY0	ASPH_MOUSE
ATP synthase mitochondrial F1 complex assembly factor 1	Q811I0	ATPF1_MOUSE
Cell surface glycoprotein gp42	P23505	GP42_RAT
DnaJ homolog subfamily B member 6	Q6AYU3	DNJB6_RAT
Dolichol-phosphate mannosyltransferase	O70152	DPM1_MOUSE
EH domain-binding protein 1-like protein 1	Q99MS7	EH1L1_MOUSE
Engulfment and cell motility protein 2	Q8BHL5	ELMO2_MOUSE
FGFR1 oncogene partner	Q4V7C1	FR1OP_RAT
Formin-like protein 2	A2APV2	FMNL2_MOUSE
Gamma-glutamylcyclotransferase	Q9D7X8	GGCT_MOUSE
Ganglioside GM2 activator	Q60648	SAP3_MOUSE
Golgin subfamily A member 2	Q62839	GOGA2_RAT
Guanine nucleotide-binding protein G(o) subunit alpha	P59216	GNAO_CRILO
Hemoglobin subunit beta	P02092	HBB_MICXA
Hepatocyte growth factor-regulated tyrosine kinase substrate	Q99LI8	HGS_MOUSE
High affinity immunoglobulin epsilon receptor subunit gamma	P20411	FCERG_RAT
Inner nuclear membrane protein Man1	Q9WU40	MAN1_MOUSE
Inositol polyphosphate 1-phosphatase	P49442	INPP_MOUSE
Kinase suppressor of Ras 1	Q61097	KSR1_MOUSE
Kynurenine--oxoglutarate transaminase 1, mitochondrial	Q08415	KAT1_RAT
Lanosterol 14-alpha demethylase	Q64654	CP51A_RAT
Mitochondrial Rho GTPase 1	Q8BG51	MIRO1_MOUSE
Mitogen-activated protein kinase 9	P49186	MK09_RAT
NADH dehydrogenase [ubiquinone] 1 alpha subcomplex subunit 12	Q7TMF3	NDUAC_MOUSE
NADH dehydrogenase [ubiquinone] 1 alpha subcomplex subunit 6	Q9CQZ5	NDUA6_MOUSE
NADH dehydrogenase [ubiquinone] 1 alpha subcomplex subunit 7	Q9Z1P6	NDUA7_MOUSE
NADH dehydrogenase [ubiquinone] 1 beta subcomplex subunit 5, mitochondrial	Q9CQH3	NDUB5_MOUSE
NADH dehydrogenase [ubiquinone] 1 subunit C2	Q9CQ54	NDUC2_MOUSE
NADH-ubiquinone oxidoreductase chain 4	P05508	NU4M_RAT
Neuroplastin	P97300	NPTN_MOUSE
N-myc-interactor	O35309	NMI_MOUSE
Normal mucosa of esophagus-specific gene 1 protein	Q5RK28	NMES1_RAT
Nuclear factor related to kappa-B-binding protein	Q6PIJ4	NFRKB_MOUSE
Nuclear RNA export factor 1	O88984	NXF1_RAT
Phosphatidylinositol 3-kinase regulatory subunit alpha	P26450	P85A_MOUSE
Procollagen galactosyltransferase 1	Q8K297	GT251_MOUSE
Programmed cell death protein 4	Q9JID1	PDCD4_RAT
Prolyl 4-hydroxylase subunit alpha-1	Q60715	P4HA1_MOUSE
Protein kinase C and casein kinase substrate in neurons 2 protein	Q9QY17	PACN2_RAT
Protein kinase C beta type	P68404	KPCB_MOUSE
Protein LSM14 homolog B	Q8CGC4	LS14B_MOUSE
Protein Wiz	O88286	WIZ_MOUSE
Putative oxidoreductase GLYR1	Q922P9	GLYR1_MOUSE
Reticulocalbin-2	Q62703	RCN2_RAT
Rho GTPase-activating protein 18	Q8K0Q5	RHG18_MOUSE
Ribosomal protein S6 kinase alpha-1	Q63531	KS6A1_RAT
Sarcoplasmic/endoplasmic reticulum calcium ATPase 3	Q64518	AT2A3_MOUSE

Sepiapterin reductase	P18297	SPRE_RAT
Serine beta-lactamase-like protein LACTB, mitochondrial	Q9EP89	LACTB_MOUSE
Serine/threonine-protein kinase TBK1	Q9WUN2	TBK1_MOUSE
Serine/threonine-protein phosphatase 2A 56 kDa regulatory subunit alpha isoform	Q6PD03	2A5A_MOUSE
Serum response factor-binding protein 1	Q66H19	SRFB1_RAT
Splicing factor 3A subunit 2	Q6AXT8	SF3A2_RAT
Squalene synthase	Q02769	FDFT_RAT
SUN domain-containing protein 2	Q8BJS4	SUN2_MOUSE
Synaptic vesicle membrane protein VAT-1 homolog	Q3MIE4	VAT1_RAT
Syntaxin-12	G3V7P1	STX12_RAT
Target of Myb protein 1	O88746	TOM1_MOUSE
Thymidylate kinase	P97930	KTHY_MOUSE
Tissue alpha-L-fucosidase	P17164	FUCO_RAT
TRAF3-interacting JNK-activating modulator	Q8C0G2	T3JAM_MOUSE
Transmembrane protein 186	Q4KLZ1	TM186_RAT
Tumor necrosis factor ligand superfamily member 10	P50592	TNF10_MOUSE
Tumor suppressor p53-binding protein 1	P70399	TP53B_MOUSE
Tyrosine-protein phosphatase non-receptor type 23 (Fragment)	O88902	PTN23_RAT
Uncharacterized protein C17orf62 homolog	Q3TYS2	CQ062_MOUSE
UPF0414 transmembrane protein C20orf30 homolog	Q5BJP5	CT030_RAT
Uracil phosphoribosyltransferase homolog	B1AVZ0	UPP_MOUSE
Vacuolar ATPase assembly integral membrane protein VMA21	Q78T54	VMA21_MOUSE
WD repeat and HMG-box DNA-binding protein 1	P59328	WDHD1_MOUSE
