# Supplementary material for: Lamellipodia and Membrane Blebs Drive Efficient Electrotactic Migration of Rat Walker Carcinosarcoma Cells WC 256
Source: PLoS One. 2016 Feb 10;11(2):e0149133. doi: 10.1371/journal.pone.0149133 (PMC4749172; doi:10.1371/journal.pone.0149133)
Supplement: S2 Table — (PDF) [file pone.0149133.s009.pdf]

S2 Table

BC					
**Parameters (±SEM)#**	**Control**	**Rac1 Inhibitor (NSC23766)**	**Cdc42 Inhibitor (ZCL278)**	**Rho Inhibitor (Rhosin)**	**ROCK Inhibitor (Y-27632)**
	**0 V/cm**				
Trajectory length [μm]	165.87 ± 5.79	177.53 ± 26.70	180.87 ± 8.63*	119.26 ± 5.30*	169.89 ± 7.58
Trajectory speed [μm/min]	5.53 ± 0.19	5.91 ± 0.89	6.03 ± 0.29*	3.98 ± 0.18*	5.66 ± 0.25
Displacement length [μm]	68.92 ± 5.16	52.96 ± 4.27*	64.88 ± 4.85	32.04 ± 4.27*	50.15 ± 5.03*
Displacement speed [μm/min]	2.29 ± 0.17	1.77 ± 0.14*	2.16 ± 0.16	1.07 ± 0.14*	1.67 ± 0.17*
Coefficient of movement efficiency CME	0.41 ± 0.09	0.31 ± 0.03*	0.36 ± 0.02	0.27 ± 0.03*	0.29 ± 0.02
Average directional cosine γ	0.06 ± 0.02	0.08 ± 0.08	0.19 ± 0.08*	-0.06 ± 0.08	-0.06 ± 0.09

LC					
**Parameters (±SEM)#**	**Control**	**Rac1 Inhibitor (NSC23766)**	**Cdc42 Inhibitor (ZCL278)**	**Rho Inhibitor (Rhosin)**	**ROCK Inhibitor (Y-27632)**
	**0 V/cm**				
Trajectory length [μm]	108.71 ± 4.33	108.39 ± 3.86	124.58 ± 4.50	104.70 ± 4.12	130.79 ± 3.36
Trajectory speed [μm/min]	0.72 ± 0.03	0.72 ± 0.02	0.83 ± 0.03	0.70 ± 0.03	0.87 ± 0.02
Displacement length [μm]	37.62 ± 2.63	33.31 ± 2.49	40.64 ± 3.62	28.70 ± 2.57*	31.42 ± 2.27
Displacement speed [μm/min]	0.25 ± 0.02	0.22 ± 0.02	0.27 ± 0.02	0.19 ± 0.02*	0.21 ± 0.02
Coefficient of movement efficiency CME	0.34 ± 0.02	0.30 ± 0.02	0.33 ± 0.02	0.27 ± 0.02*	0.25 ± 0.02*
Average directional cosine γ	0.05 ± 0.01	0.06 ± 0.07	0.03 ± 0.08	-0.08 ± 0.08	-0.06 ± 0.08

#Definitions of parameters and details of the statistics are given in Materials and Methods

*Statistically significant vs. Control ($p<0.05$)
