# Supplementary material for: Lamellipodia and Membrane Blebs Drive Efficient Electrotactic Migration of Rat Walker Carcinosarcoma Cells WC 256
Source: PLoS One. 2016 Feb 10;11(2):e0149133. doi: 10.1371/journal.pone.0149133 (PMC4749172; doi:10.1371/journal.pone.0149133)
Supplement: S1 Text — (DOCX) [file pone.0149133.s010.docx]

**Proteomic analyses**

***Filter Aided Sample Preparation for LC-MS/MS analysis***

Supernatant was diluted by 8 M urea in 50 ammonium bicarbonate (300 μl), reduced with DTT (final concentration was 50 mM, 15 min.) and applied on the 30 kDa cut-off filter (Vivacon 500, Sartorius Stedim, Germany). After centrifugation (14,000 × g, two-times 15 min, RT) the proteins were washed with 200 μl of 8M urea and centrifuged (14,000 × g, two-times 45 min, RT). The proteins were alkylated with iodoacetamide (final concentration was 0.1 mg/ml in 8 M urea, 20 min., in the dark). The samples were centrifuged and subsequently washed three times with 8 M urea (14,000 × g, two-times 25 min, RT) and four times with 50 mM ammonium bicarbonate (14,000 × g, two-times 20 min, RT). After the last centrifugation step, 75 μl of ammonium bicarbonate containing 2.5 μg of trypsin was added. After on-filter protein digestion (overnight, 37 °C) the resulting peptides were spin down (14,000 × g, 30 min, 25°C) and the filter unit was washed two times by 40 μl of ammonium bicarbonate and additionally with 50 μl 0.5 M NaCl (14,000 × g, 30 min, 25°C). Afterwards 2 μl of 100% TFA was added, peptide samples were centrifuged (35,000 × g, 20 min, 4°C) and moved into vial insert prior to LC-MS/MS analysis.

The peptide content was estimated by UV light spectral density at 280 nm (Nano-Drop, Thermo) using an extinctions coefﬁcient of 1.1 of 0.1% (g l–1) solution that was calculated on the basis of the frequency of tryptophan and tyrosine in vertebrate proteins [1]. 1.5 µg of peptides were loaded on 50 cm column.

***Liquid chromatography and tandem mass spectrometry (LC-MS/MS)***

Peptides were analyzed by mass spectrometry (MS) using an UltiMate 3000RS LC nanoSystem (Dionex) coupled with Q-Exactive mass spectrometer (Thermo Fisher Scientific, Bremen, Germany) with DPV-550 Digital PicoView nanospray source. Peptides were injected on a C18 precolumn (Acclaim PepMap Nano trap Column) using 2% acetonitrile with 0.05% TFA as a mobile phase, and further separated on a 50 cm × 75 μm RP column (Acclaim PepMap 75 μm 100 Å Nano Series TM Column) with gradient 2–40% ACN in 0.05% FA for 360 minutes. The electrospray voltage was 2.2 kV and the ion transfer tube temperature was 250 °C. The Q-Exactive was operated in data dependent mode using “fast method” based on [2] with slightly modifications. Full MS scans were acquired in the Orbitrap mass analyzer over m/z 300–2000 range with resolution 70,000 (at m/z  200). The target value was 1.00E+06. The top twelve most intense peaks with charge state ≥ 2 were fragmented in the HCD collision cell normalized collision energy of 27%, (the isolation window was 1.2 m/z). Tandem mass spectrum was acquired in the Orbitrap mass analyzer with resolution 17,500 at m/z 200. The target value was 5.00E+05. The ion selection threshold was 1.10E+05 counts, and the maximum ion accumulation times for the survey scan and the MS/MS scans were 120 ms and 60 ms respectively, dynamic exclusion was set to 30 s.

**References**

1. Zhuang, Y, Ma F, Li-Ling J, Xu, X, Li,Y. Comparative analysis of amino acid usage and protein length distribution between alternatively and non-alternatively spliced genes across six eukaryotic genomes. Mol Biol Evol. 2003; 20; 1978–1985.

2. Kelstrup CD, Young C, Lavallee R, Nielsen ML, Olsen JV. Optimized fast and sensitive acquisition methods for shotgun proteomics on a quadrupole Orbitrap mass spectrometer. J Proteome Res.2012; 11: 3487.
